# Minimal physicalism as a scale-free substrate for cognition and consciousness

**DOI:** 10.1093/nc/niab013

**Published:** 2021-08-02

**Authors:** Chris Fields, James F Glazebrook, Michael Levin

**Affiliations:** 1 23 Rue des Lavandières, 11160 Caunes Minervois, France; 2 Department of Mathematics and Computer Science, Eastern Illinois University, 600 Lincoln Ave, Charleston, IL 61920 USA; 3 Department of Mathematics, Adjunct Faculty, University of Illinois at Urbana–Champaign, 1409 W. Green Street, Urbana, IL 61801, USA; 4 Allen Discovery Center, Tufts University, 200 College Avenue, Medford, MA 02155, USA

**Keywords:** active inference, aneural systems, basal cognition, classical computation, integrated information, memory, quantum computation, self-representation, state broadcasting

## Abstract

Theories of consciousness and cognition that assume a neural substrate automatically regard phylogenetically basal, nonneural systems as nonconscious and noncognitive. Here, we advance a scale-free characterization of consciousness and cognition that regards basal systems, including synthetic constructs, as not only informative about the structure and function of experience in more complex systems but also as offering distinct advantages for experimental manipulation. Our “minimal physicalist” approach makes no assumptions beyond those of quantum information theory, and hence is applicable from the molecular scale upwards. We show that standard concepts including integrated information, state broadcasting via small-world networks, and hierarchical Bayesian inference emerge naturally in this setting, and that common phenomena including stigmergic memory, perceptual coarse-graining, and attention switching follow directly from the thermodynamic requirements of classical computation. We show that the self-representation that lies at the heart of human autonoetic awareness can be traced as far back as, and serves the same basic functions as, the stress response in bacteria and other basal systems.

## Introduction

The starting point for sciences of consciousness and cognition has traditionally been human consciousness and cognition. The Cartesian presumption against nonhuman consciousness and cognition remained strong enough, even just two decades ago, that prominent researchers found it necessary to argue in print that nonhuman animals and even human infants experienced perception and interoception and engaged in intentional actions (e.g. Panksepp 2005; Trevarthen 2010; Rochat 2012). Those days are thankfully behind us. While researchers in comparative cognition may debate the levels of complexity, compositionality, or stimulus-independence an information-processing system must have to be regarded as “cognitive” (see Bayne *et al.* 2019 for a recent snapshot), big-brained creatures that learn readily and display flexible behavior in complex environments are now regularly regarded as cognitive systems that are aware of both their environments and their own bodily states, even if they are birds (e.g. Güntürkün and Bugnyar 2016; Nieder *et al.* 2020) or cephalopods (e.g. Mather 2019; Schnell *et al.* 2020). To insist that such creatures altogether lack phenomenal consciousness, that they are incapable of experiencing pain and likewise experience nothing whatsoever while hunting, improvising tools, or engaging in mating displays as a doctrinaire Cartesian presumably would, seems by current standards chauvinistic or even perverse.

Characterizing birds and cephalopods, and for that matter other mammals and even other people, as phenomenally conscious begs, of course, the philosophical questions of what phenomenal consciousness is ontologically, and of how it relates to observable structures, functions or behaviors. These questions constitute the “hard problem” (HP) of consciousness (Chalmers 1996; *cf*. Dietrich and Hardcastle 2005; Dietrich *et al.* 2021). We agree with Klein and Barron (2020) that the HP is not a problem to be solved, but rather a set of intuitions to be overcome. The questions of interest regarding birds, cephalopods, other mammals, and other people are not, as far as we are concerned, whether or why, in some philosophical sense, they are capable of experience, but rather: (1) what any particular organism or other system of interest is aware of, and (2) how its awareness drives its cognition. Our interest here is not, moreover, in what any particular experience is like for any given system, but in whether it is like something or other; to employ a common example, it is not the particular characters of red or green qualia that are of interest (see e.g. Hacker 2002 for an argument that no such particular characters even exist), but whether some difference between what we, as 3rd party observers, call “red” and “green” is experienced by the system of interest, and whether this difference has some effect on what the system does. Hence we regard consciousness as a (potential) property that a system may have, independently of any details about what it experiences: it is enough, in our view, that it experiences something, that some experience occurs (see Lamme 2018 for a discussion of some of the subtleties that this “binary” view elides). We will use the terms “awareness” and “consciousness” to mean the capacity for or capability of having phenomenal experiences, however basic or minimally structured, and will focus exclusively in what follows on the “what” and “how” questions above.

A well-established literature extends the concepts of consciousness—the capability of having phenomenal experiences, however basic or minimally structured—and cognition to phylogenetically basal systems, including free-living or facultatively communal unicells, whether pro- or eukaryotic (Maturana and Varela 1980; Pattee 1982; Stewart 1996; di Primio *et al.* 2000; Lyon 2015; Baluška and Levin 2016; Baluška and Reber 2019, 2021; Levin 2019, 2020; Fields and Levin 2020a; Lyon 2020; Baluška *et al.* 2021), plants (Calvo and Kiejzer 2009; Gagliano and Grimonprez 2015; Baluška *et al.* 2018; Debono and Souza 2019; Calvo *et al.* 2020), and aneural or lower (than mammals, birds, or cephalopods) complexity neural metazoa, particularly flatworms (Shomrat and Levin 2013; Inoue *et al.* 2015; Levin 2020) and insects (Menzel 2012; Perry *et al.* 2017; Pfeffer and Wolf 2020). Like the extension of these concepts from humans to nonhuman mammals and then to big-brained nonmammals, this extension to more basal organisms was initially motivated by observations of communication, learning, and behavioral flexibility, and by functional similarities between the mechanisms supporting information processing and learning in basal systems and in more complex systems such as mammals. Both molecular and bioelectric mechanisms of cellular information processing, memory, communication, and error correction are, in particular, evolutionarily ancient and conserved across phylogeny (Fields and Levin 2018, 2020b; Fields *et al.* 2020; Levin 2020). Neither the specialization of some epithelial cells for communication, i.e. as neurons (Nickel 2010; Arendt *et al.* 2015; Burkhardt and Sprecher 2017) nor the gradual elaboration of brains (Holland *et al.* 2013; Liebeskind *et al.* 2017) introduce fundamentally novel molecular or bioelectric mechanisms. Hence while claims of specific kinds of experience, e.g. pain in plants (Draguhn *et al.* 2020), are controversial, there is no evident biological discontinuity below which organisms can clearly be viewed as completely unaware of their bodily states or their environments, i.e. as utterly lacking phenomenal consciousness. Nor is it obvious that supra-organismal systems utterly lack phenomenal consciousness (Friedman and Søvik 2021). Indeed, the criteria for “having experiences” may be as vague, general, and extensible to non-Terrestrial or even artificial systems as plausible criteria for “life” are (e.g. Bartlett and Wong 2020; but see also Mariscal and Doolittle 2020 for an argument that such “definitionism” is scientifically pointless).

With the development of more sophisticated, biologically motivated theoretical models of consciousness and cognition, the functional similarities between information processing in basal and in more elaborated systems have become even more evident. It is now well-known that signal transduction and gene regulatory networks in single cells share the scale-free, small-world topology (Agrawal 2002; Barabási and Oltvai 2004; Schlitt and Brazma 2007) found ubiquitously in nervous systems (Sporns and Honey 2006; Hagmann *et al.* 2008; Shanahan 2012; Sporns 2013) and even in social and technological networks (Watts 1999; Barabási 2016; but see Broido and Clauset 2019 for evidence that some biological and social networks are small-world but not strictly scale-free). Networks with high fan-in/high fan-out “bow-tie” topology (Wang *et al.* 2016; Niss *et al.* 2020), in particular, implement state-broadcasting functions analogous, at the cellular level, to the broadcasting functions of long-distance connections between network hubs in global neuronal workspace (GNW) models (Dehaene and Naccache 2001; Baars and Franklin 2003; Wallace 2005; Baars *et al.* 2013; Dehaene *et al.* 2014; Mashour *et al.* 2020). Signal transduction and gene regulatory networks are, even when they contain bow-tie nodes, characterized by multiple, typically cross-modulating feedback loops that enable both behavioral plasticity and learning (e.g. Brun-Usan *et al.* 2020); hence they have positive integrated information Φ and render their minimal containing systems conscious by the criteria of Integrated Information Theory (IIT; Tononi 2008; Oizumi *et al.* 2014; Tononi and Koch 2015). As the locus of molecular, thermodynamic, and bioelectric exchange with the environment, the cell membrane implements a Markov Blanket (MB) that renders its interior conditionally independent of its exterior (Pearl 1988; Clark 2017); this allows the cell to be described as a Bayesian active inference system (Friston 2010, 2013; see also Cooke 2020 for a variation on this approach). The utility of this Bayesian approach has been demonstrated in simulation models of cell–cell communication driving morphoghenesis (Friston *et al.* 2015; Kuchling *et al.* 2020). These cross-scale similarities motivate a hypothesis that consciousness and cognition are scale-free phenomena that characterize all living systems (Levin 2019, 2020; Fields and Levin 2020a).

If consciousness and cognition are scale-free phenomena, we can expect them to be supported by common, scalable mechanisms that can be investigated in whatever systems permit the most straightforward theoretical and experimental approaches. Phylogenetically basal organisms, *in vitro* preparations, and synthetic constructs (e.g. Kriegman *et al.* 2020) provide obvious advantages of manipulability and environmental control. Studies of basal systems are, moreover, especially effective in overcoming the intuitions that give rise to the HP, as they allow the mechanisms via which single cells and relatively simple multicellular organisms navigate their environments—mechanisms that they share with most of our cells, and with us as organisms—to be investigated in detail. A theoretical framework suitable for such systems should be scalable, provide a full suite of formal capabilities, and make as few assumptions as possible. It should, in particular, make no scale-dependent architectural assumptions.

Here, we provide a straightforward construction of fundamental, scale-free features of consciousness and cognition within a generic description of system-environment information exchange as bipartite physical interaction (Fields and Glazebrook 2020a; Fields and Marcianò 2020a,b). We term this description “minimal physicalism” (MP) as it makes no assumptions about classical computational architecture, in particular, no assumptions about network architecture (Broido and Clauset 2019), and no physical assumptions beyond those of quantum information theory (Nielsen and Chuang 2000 provide a standard introduction). At the level of molecular interactions at the Angstrom, femtosecond (Å, fs) scale of molecular dynamics calculations (Zwier and Chong 2010; Vlachakis *et al.* 2014), biological systems are quantum systems, and biological information processing is quantum computation: cellular energy budgets of both prokaryotes and eukaryotes fall orders of magnitude short of the power required to maintain classical states of just protein conformation and localization at this scale (Fields and Levin 2021), despite the massive consumption of ATP by big-brained eukaryotes such as humans (Ueno *et al.* 2005; Okuno *et al.* 2011). Hence cellular information processing cannot be entirely, or even primarily classical, the experimental difficulties (Cao *et al.* 2020) of unambiguously observing quantum coherence in biological systems notwithstanding.

As we show below, the thermodynamic requirements of classical computation by quantum systems have profound consequences for cellular, and hence for organismal, awareness and information processing. In general, basing MP on the physical assumptions of quantum information theory has significant empirical consequences that extend far beyond a mere rejection of dualism. These include:

1. MP is completely scale-free, applying in the same form to interactions between molecules, cells, tissues, organisms, social groups, or ecosystems and their respective environments. Hence it predicts common mechanisms that can be probed empirically at any scale. It makes no assumptions about the structure or dynamics of the environment, at any scale, beyond its being a physical system.
2. MP requires every property of either itself or the environment to which an organism or other living system is differentially sensitive to be specified explicitly in terms of the information processing employed by the organism or system to detect and respond to that property. This includes properties often taken for granted, such as the existence of external objects or their embedding in three-dimensional (3d) space, and applies regardless of whether detection and/or response is recorded to memory or “reportable” via any specific assay.
3. MP treats information and energy as formally equivalent, explicitly enforces conservation of energy, and hence requires the thermodynamics of classical computation (Landauer 1961; Bennett 1982) to be represented explicitly. It requires the thermodynamic cost of memory to be accounted for at every scale, and automatically enforces a metabolism—cognition tradeoff.

Information processing functions, including writing to or reading from a classical memory, are specified in this framework using the scale- and implementation-independent, category-theoretic language of Barwise-Seligman (“token” – “type”) classifiers and their associated infomorphisms (Barwise and Seligman 1997; see Fields and Glazebrook 2019a for review and example applications). This generic specification language has been previously employed to characterize human object and event categorization using both abstraction and mereological hierarchies (Fields and Glazebrook 2019b) and human problem solving within a GNW context (Fields and Glazebrook 2020b). As it requires the reference frames employed to give internal, e.g. perceptual or motor, representations operational meaning to be explicitly specified, this language requires all semantic hypotheses to be formulated in explicit, experimentally accessible molecular or network-theoretic terms.

We outline the main components of MP in the next section, keeping formalism to the minimum and relying on previous work for details. We show that MBs and a notion of actionable or “meaningful” (Bateson 1972; Roederer 2005) information emerge naturally in an MP framework. We then discuss predictions of MP regarding perception, memory, and attention, providing examples primarily from basal systems for illustration. We show that requirements for coarse-graining and attention switching emerge naturally from the thermodynamics of classical computation. We then consider resource-usage monitoring as a requirement of homeo- or allostasis, and the emergence of a “self” as a representation of resource usage.

## Components of MP: Information Exchange, Markov Blankets, and Reference Frames

### Physical interaction is information exchange

The re-interpretation of physics as an information theory was initiated by Feynman (1982) and Wheeler (1990), but has its roots in the work of Boltzmann (1995), who first recognized that reducing uncertainty has an energetic cost, and Shannon (1948), who linked uncertainty reduction with communication. It is deeply rooted in Wheeler’s (1983) idea of “observer-participancy”: that information can only be obtained by intervention, i.e. by actively posing a question (see Mermin 2018; Müller 2020 for recent, somewhat philosophical discussions). Physics is intrinsically quantum due to the quantization of obtainable information into bits: answers to yes/no questions (Wheeler 1983). The basic formalism of this quantum information theory is surprisingly simple, and rests on only the two assumptions specified below.

The simplest physical interactions are bipartite: some physical, i.e. quantum system *A* (conventionally called “Alice”) interacts with some other system *B* (“Bob”). We can therefore write:

(1) U=AB

or more explicitly, in terms of Hilbert spaces:

(2) $$H_{U}=H_{A}\;⊗H_{B}$$

where *U* is the joint system (“universe”) comprising *A* and *B* and ⊗ is the Hilbert-space tensor product. The dynamics of the joint system is completely described by a Hamiltonian operator:

(3) HU= HA+ H B+ HAB

where *H_A_* and *H_B_* are the internal or “self” interactions of *A* and *B* respectively and *H_AB_* is their mutual interaction. The operator *H_U_* has units of energy and drives state change via the Schrödinger equation:

(4) $$i\hbar \left(\partial /\partial t\right)\;\left|U\left(t\right)>=H_{U}\;\right|U\left(t\right)>$$

where *i* = √-1, *ℏ *=* h*/2π, *h* is Planck’s constant, and |*U*(*t*)> is the time-dependent state of *U*. The mutual interaction *H_AB_* describes Alice’s observations of and actions on Bob, and Bob’s observations of and actions on Alice. The internal interactions *H_A_* and *H_B_* describe Alice’s and Bob’s internal state changes.

The above descriptions are completely generic and characterize any bipartite interaction between quantum systems (Nielsen and Chuang 2000). As a quantum system is simply a collection of physical degrees of freedom, this formalism makes, in particular, no assumptions about implementation: it applies equally to matter (e.g. molecules) or fields (e.g. electromagnetic fields as in McFadden 2020). We now impose two assumptions:

1. We require the dimension of the joint system *U* to be finite. This limits both *A* and *B* to finite energy resources and *H_AB_* to finite resolution.
2. We require the joint state |*U*> = |*AB*> to be separable, i.e. |*U*> = |*AB*> = |*A*>|*B*>. Equivalently, *A* and *B* are not entangled. In this case, *H_AB_* specifies the entire dependence of *A* on *B* and vice-versa, and we can talk about the state |*A*> of *A* and the state |*B*> of *B* independently.

With these two assumptions, the interaction *H_AB_* can be written, without loss of generality, as:

(5) $$H_{AB}\left(t\right)=\beta^{k}\;k_{B}\;T^{k}\;\Sigma_{i}\;\alpha_{i}^{k}\left(t\right)M_{i}^{k}$$

where *k = A* or *B*, *i *=* *1 … *N* for finite *N*, *k_B_* is Boltzmann’s constant, *T^k^* is *k’*s temperature, β^*k*^ ≥ ln 2 is an inverse measure of *k’*s average thermodynamic efficiency that depends on the internal dynamics *H_k_*, the α*^k^_i_*(*t*) are *N* real functions such that, for any macroscopic time interval Δ*t*:

(6) $$\Sigma_{i}\;{\int \Delta }_{t}\;\alpha_{i}^{k}\left(t\right)\;dt=\Delta t$$

and the *M^k^_i_* are *N* Hermitian operators with binary eigenvalues +1 or -1 that can be regarded as “questions to Nature” with yes/no answers [Wheeler 1983; see Fields and Glazebrook 2020a; Fields and Marcianò 2019, 2020b for details on Equations (5) and (6) and their interpretation]. The requirement that β^*k*^ ≥ ln 2 in Equation (5) enforces Landauer’s principle of the finite per-bit cost of irreversibly acquiring an observational outcome (Landauer 1961; Bennett 1982), while Equation (6) imposes a normalization condition, equivalent to the requirement that the probability of acquiring some outcome whenever a measurement is made is unity. As we discuss below, Equations (5) and (6) are the only physical requirements needed to describe both free-energy exchange and information transfer in a generic bipartite interaction between finite quantum systems in a separable joint state. We can write Equation (5) in English as:

Physical interaction=(thermodynamics)×(yes/no questions)

This is the conceptual heart of quantum information theory. It, like Equations (5) and (6), applies at any scale.

### Interactions induce MBs

**Figure 1.**
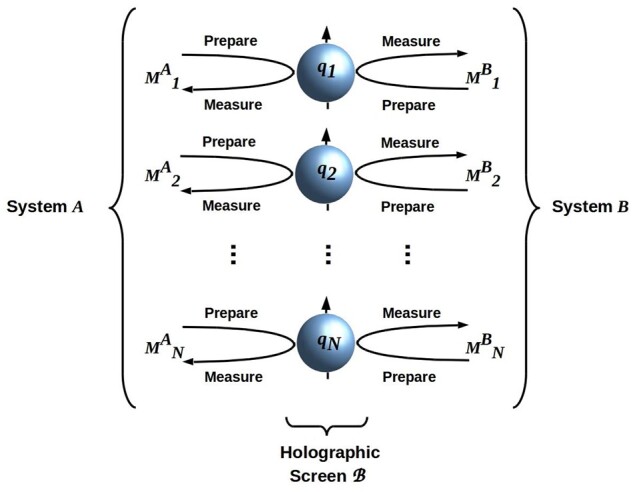
The interaction *H_AB_* specified by Equation (5) can, without loss of generality, be implemented by alternating preparation and measurement of *N* mutually noninteracting qubits. Each cycle of interaction has two phases: first, *A* prepares the *N* qubits and then *B* measures them, then *B* prepares the *N* qubits and *A* measures them. The qubit array defines a holographic screen B separating *A* from *B*. This screen enforces conditional independence between *A* and *B*, and hence functions as a MB. There is no source of classical noise in the interaction; however, there is quantum noise, and hence potential classical communication error, whenever *A* and *B* employ different reference frames (e.g. different *z*-axes) to prepare and measure the qubits. Adapted from Fields and Marcianò (2020b); CC BY license.

The complete generality of Equation (5) allows us to view any interaction between two finite quantum systems in a separable joint state as classical communication: the exchange of some finite number of finite bit strings during any finite time interval Δ*t*. The interaction can be depicted as in Fig. 1: *A* and *B* can be regarded as alternately preparing, and then measuring *N* independent, mutually noninteracting quantum bits (qubits), e.g. photon polarizations, electron spins, or any other systems having two clearly distinguishable values of a manipulable state variable. In each cycle of interaction, *A* prepares the *N* qubits and then *B* measures them; the roles then reverse, with *B* preparing and *A* measuring. As *N* becomes large, the interaction may appear “continuous” at macroscopic scales, but remains constrained to finite resolution, and hence a finite bit rate, by the restriction to finite thermodynamic resources built into Equation (5). The time required for *A* to prepare, and then *B* to measure (or vice versa), the *N* systems can be regarded as the smallest possible “macroscopic” and hence effectively classical time interval (see Fields and Glazebrook 2020 for an explicit construction of this interval). The collection of all *N* qubits encodes, at each such minimal macroscopic time, one *N*-bit string specifying one of the 2^*N*^ eigenvalues of *H_AB_*; each cycle of interaction is thus an exchange of *N*-bit strings, each one encoding an (in general different) eigenvalue.

The encoding of eigenvalues by qubits shown in Fig. 1 satisfies the requirements for a holographic encoding: the only information about *B* available to *A*, or vice versa, is the sequence of eigenvalues of *H_AB_* encoded by the qubit array. Hence we can consider the qubit array as a holographic screen B separating *A* from *B*. Any such holographic screen defines a classical communication channel, i.e. a channel via which *A* and *B* exchange finite bit strings (Fields and Marcianò 2020b; Fields *et al.* 2021). As no other system interferes with this channel, it is free of classical noise. However, it is characterized by quantum noise whenever *A* and *B* employ different reference frames (e.g. different *z*-axis reference frames in the case of qubits implemented by electron spins) to prepare and measure the qubits. The effect of such quantum noise is to impose a classical probability distribution *Prob*(measured|prepared) on each qubit; i.e. its observable effect is indistinguishable from classical noise.

If we now consider the internal (quantum) states |*A*> and |B> and the states |B> of the qubit array forming the holographic screen between them, we see that |*A*> affects |*B*> only via its effect on |B>, i.e. only via the action of *H_AB_*. This is, as noted above, a consequence of separability: separability implies conditional independence. Conditional independence of |*A*> from |*B*> and vice-versa is, however, also the MB condition (Pearl 1988; Friston 2013; Clark 2017); hence we can view B as an MB. Indeed, the generalization of the holographic principle stated by Equation (5) and illustrated in Fig. 1 implies that any holographic screen defines a classical information channel that functions as an MB, and vice versa (Fields and Marcianò 2020a; Fields *et al.* 2021).

As discussed in Friston (2013), MBs are ubiquitous features of living systems, and are indeed what distinguish living systems from their environments (see also Friston *et al.* 2015; Kuchling *et al.* 2020; Fields and Levin 2020c). Indeed MBs are ubiquitous features of all physical systems with ergodic behavior (Friston 2019). The MB concept is scale-free; Rubin *et al.* (2020) show, e.g. that it applies to Earth’s biosphere as a whole. The cell membrane, and in eukaryotic cells, organelle membranes, implement the most fundamental biological MBs. The blanket states of these MBs comprise the states of the embedded receptors, channels, and other exchange molecules, together with the permeability, elasticity, and other properties of the membrane itself. The existence of the MB enables the cell to have a well-defined state, conditionally independent of its environment; hence it enables homeostatic/allostatic processes. The information about the environment available to the cell, and vice-versa, is limited to that encoded by the state of the MB. A similarly strong conditional independence result has been obtained via evolutionary game theory by considering generic systems subjected to competition for environmental resources (Hoffman *et al.* 2015; Prakash *et al.* 2020a,b).

The central importance to the cell of the MB implemented by its membrane strongly suggests that enclosure by a membrane was the fundamental requirement for the origin of life (Friston 2013; Fields and Levin 2020c). What was enclosed by the MB/membrane of the last universal common ancestor (LUCA) represents the initial condition and oldest memory (Fields and Levin 2018) of all subsequent life. Viewed from an informational perspective, the state space of LUCA’s membrane-enclosed cytoplasm, including its nucleic-acid, protein, and small-molecule components, specifies the initial information about the environment available prior to membrane enclosure and hence physical separation from the environment. While the cytoplasmic conditions of later cells have diverged from these initial conditions, sometimes radically, they remain a fixed constraint with which all paths of divergence must be consistent. Here, the classical meaning of “separation” from the environment as the presence of a physical barrier accords with its quantum-theoretic meaning of state distinguishability. We can, indeed, see the former as implied by the latter.

### Actionable information is encoded by quantum reference frames

The information that transits the cell membrane, and is thereby encoded on the MB implemented by the membrane, is actionable or meaningful to the cell: it “makes a difference” to what the cell does (Bateson 1972; Roederer 2005). When the cell’s interaction with its environment is represented as measurement as in Equation (5) or Fig. 1, what renders the information meaningful becomes clear: meaning requires measurement with respect to some reference frame (Fields and Levin 2020a; Fields *et al.* 2021). Viewed abstractly, a reference frame is a value, or more generally a vector, from which deviation is detectable. Consider, e.g. measuring the states of an array of qubits implemented by electron spins. Determining whether the spin of a qubit is up (+1) or down (–1) requires a reference frame that defines a particular *z*-axis, a vector in 3d space. Such a vector can be defined by, e.g. a Stern-Gerlach apparatus oriented in some particular way relative to the Earth’s gravitational field. Only the presence of a common reference frame allows multiple measurements to be compared, and hence makes any differences between them meaningful. Sequential measurements of electron spin with respect to a randomly varying *z*-axis merely yield noise.

While in classical physics a reference frame is an abstraction that can be completely described to arbitrary precision, this is not true in quantum theory (Bartlett *et al.* 2007). Quantum theory requires all reference frames to be implemented by physical systems, but forbids the description of any physical system to arbitrary precision. Hence quantum reference frames (QRFs) cannot be completely described to arbitrary precision; they encode, in virtue of their physical implementation, “unspeakable” (Bell 1987) or “nonfungible” (Bartlett *et al.* 2007) information. This is even true of meter sticks, the lengths of which become uncertain as the energies required to probe them become comparable to the binding energies of nucleii. It is much more obviously true of complex systems such as Stern-Gerlach apparatus or atomic clocks.

The intrinsically nonclassical nature of QRFs has some surprising consequences. Transferring a QRF from one observer to another requires transferring the physical implementation; transferring a finite bit string is provably insufficient (see Bartlett *et al.* 2007 for proof and examples). Any such transfer requires, moreover, that sender and receiver already share a QRF sufficient to identify the transferred system (Fields and Marcianò 2019). We can, therefore, without loss of generality regard all QRFs available to any observer, including any organism, as physically implemented by the dynamical structure of that system, i.e. by its internal Hamiltonian. All QRFs are, therefore, quantum informational processes, i.e. quantum computations, implemented by physical systems that act as observers. The computations that implement QRFs are, as we will see below, key to understanding both what organisms are aware of and how their awareness drives their cognition. They are, therefore, the central components of any scientific theory of consciousness and cognition that is mathematically consistent with quantum information theory.

The Che-Y system employed by bacterial chemotaxis, e.g. in *Escherichia coli*, provides a simple example of a biological QRF. If the concentration [Che-Y-P] of phosphorylated Che-Y is high, the flagellar motor spins counter-clockwise and the bacterium swims in a straight line; if [Che-Y-P] is low, the motor spins clockwise and the bacterium tumbles (Wadhams and Armitage 2004; Micali and Endres 2016). “High” and “low” concentrations of phosphorylated Che-Y are defined with respect to some default concentration ratio [Che-Y-P]/[Che-Y] of phosphorylated to unphosphorylated forms, which in turn depends on the balance of relevant kinases and phosphorylases. The default ratio [Che-Y-P]/[Che-Y] is the QRF for the Che-Y system; it fixes a particular value at which behavior switches from straight-line swimming to tumbling. Whether the measured [Che-Y-P]/[Che-Y] at any given time is above or below this fixed value effectively determines whether a stimulus is approached, i.e. is considered “good” by *E. coli* or avoided, i.e. considered “bad.”

In any electrically excitable cell, the resting membrane potential *V*^0^_*mem*_ is a QRF for polarization: values of *V_mem_* > *V*^0^_*mem*_ are depolarized, while values of *V_mem_* < *V*^0^_*mem*_ are hyperpolarized. The setting of this reference frame is critical to cell behavior in both neurons and nonneural cells. In the planarian *Dugesia japonica*, altering *V*^0^_*mem*_ of blastomere cells by blocking cell communication via gap junctions during regeneration can effect a homeotic transformation of the posterior from tail morphology to head morphology, including a complete functional brain (Oviedo *et al.* 2010; Durant *et al.* 2017). In both neurons and electrically excitable plant cells, blocking gap junctions via anesthetics induces cell quiescence (Baluška *et al.* 2016; Gremiaux *et al.* 2014; see also Kelz and Mashour 2019 for a general discussion of anesthetic effects across phylogeny).

The quantum theory of complex, macroscopic systems is generally intractable; hence investigating the general properties of biological QRFs requires an abstract, scale-free specification language. The category-theoretic formalism of Channel Theory, developed by Barwise and Seligman (1997) to describe networks of communicating information processors, provides a suitably general language for specifying the functions of QRFs without any specific assumptions about their implementation. The information processing elements in this representation are logical constraints termed “classifiers” that can be thought of as quantum logic gates; they are connected by “infomorphisms” that preserve the imposed constraints (numerous application examples, mainly in computer science, are reviewed in Fields and Glazebrook 2019a). Combinations of these elements are able to implement “models” in the sense of good-regulator theory (Conant and Ashby 1970). Suitable networks of classifiers and their connecting infomorphisms, e.g. provide a generalized representation of artificial neural networks (ANNs) and support standard learning algorithms such as back-propagation. Networks satisfying the commutativity requirements that define “cones” and “cocones” (“limits” and “colimits,” respectively, when these are defined) in category theory (Goguen 1991) provide a natural representation of both abstraction and mereological hierarchies (Fields and Glazebrook 2019b) and of expectation-driven, hierarchical problem solving, e.g. hierarchical Bayesian inference and active inference (Fields and Glazebrook 2020b,c). Commutativity within a cone—cocone structure, in particular, enforces Bayesian coherence on inferences made by the structure; failures of commutativity indicate “quantum” context switches (Fields and Glazebrook 2020c).

## Predictions of MP: Awareness, Memory, and Attention

### Awareness of X requires a QRF for X

Theories of consciousness typically assume that the agent of interest is embedded in an environment that has observer-independent features and contains observer-independent objects. The visual system, e.g., is often described as using “inverse optics” to compute an “observer-independent” 3d layout from a 2d image (e.g. Pizlo 2001). Assuming observer-independent, ontological “givens” both restricts the agent of interest to some subset of our (typically assumed *a priori*) ontology and risks under-predicting the agent’s computational requirements. It makes von Uexküll’s (1957) question of defining the organism’s *umwelt*, and hence Nagel’s (1974) “what is it like?” question harder, and makes the Cartesian response that most organisms are aware of nothing, and hence have no *umwelt* easier.

Quantum theory itself forces MP to reject environmental “givens” on purely formal grounds. The Hilbert space H_*B*_ in Equation (2) and internal Hamiltonian *H_B_* in Equation (3) can be decomposed in any arbitrary way without affecting the interaction *H_AB_* at all (Fields 2012, 2016); hence *H_AB_* can communicate no information to *A* about any such decompositions. This is the case for any bipartite interaction, and hence any interaction described by Equation (5), regardless of the physical makeup of the interacting components. It is the conditional independence between the inside and outside of an MB expressed in more fundamental physical terms.

**Figure 2.**
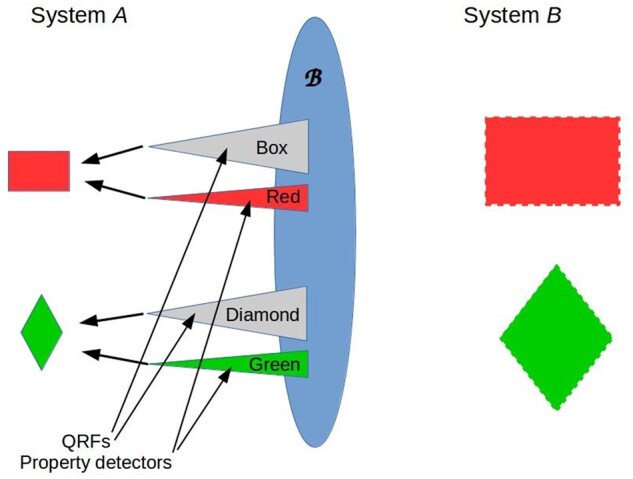
Simplified cartoon of feature or object perception in MP. The depicted relationship between *A* and *B* is topological: they are separated by the boundary B. There is no implied geometry, and the interaction is bipartite: there is no third system “outside” *U = AB* with which *A* or *B* interact. Features or objects “embedded” in the environment *B* are perceptible only by systems *A* equipped with QRFs and property detectors that render the features/objects both detectable and meaningful, and are defined only relative to such systems; this lack of observer-independent ontology is indicated here by dashed boundaries. Triangles within *A* suggest the form of classifier cocones when drawn as diagrams (Fields and Glazebrook 2019a,b; 2020a,b,c); arrows indicate binding operations. The analogy with mammalian visual feature detection is obvious; see Fields and Glazebrook (2019b) for a detailed formal construction.

By rejecting any *a priori* ontology for the environment, MP requires any perceived ontology to be fully supported by the information-processing capabilities of the perceiver. In particular, any perceived feature of, or perceived object embedded in, the environment *B* of any agent of interest *A* must be rendered both detectable and meaningful by a QRF implemented by *A*. This is illustrated in cartoon form in Fig. 2: detecting an environmental feature (a box) with a property (a color) requires a QRF for “box-ness” and a specific detector for the color. The box-detecting QRF encodes those properties (reference state observables) that boxes must have to qualify as boxes; hence it picks out boxes, and only boxes, against the general background of *B*. Informally, the QRF can be thought of as an attractor in an “interpretation space” associated with the system implementing the QRF. The property detector determines the value of some “pointer” observable that characterizes all systems that qualify as boxes; here, the box’s color. In quantum-theoretic language, identifying a bounded system (feature or object) having some specific property “decoheres” *B* relative to *A* (see Fields and Glazebrook 2020a; Fields and Marcianò 2020b for formal analysis). It is important to emphasize that *A*’s perceptual and cognitive capabilities have no effect on the physical state |*B*>, and in particular do not render it separable. The features or objects that *A* detects in *B* are strictly relative to *A*, not “objective” in any observer-independent sense (again see Mermin 2018; Müller 2020 for discussion from a purely quantum-theoretic perspective).

Specifying “objects of awareness” entirely in terms of QRFs, with no claims of observer-independent ontology, renders typical formulations of the “combination problem” ill-posed. Combination is well-defined for QRFs: two QRFs implemented by a single system can be combined if, but only if, all of the operators (i.e. the Barwise-Seligman infomorphisms) involved mutually commute (Fields and Glazebrook 2020c). This is the “combination” implemented by standard binding processes, e.g. feature or feature-motion binding during object categorization (Fields and Glazebrook 2019b). “Combination” of QRFs implemented by different systems is, on the hand, strictly undefined: the measurement operators implemented by different systems act on different environments, each of which by definition contains (at least partially) the other system. Hence what Chalmers (2017) calls the “quality” and “structure” combination problems cannot be coherently posed in MP. What Chalmers calls the “subject” combination problem is solved, for living systems, by their environments; systems that cannot maintain homeostasis/allostasis do not survive, and are unlikely to assemble at all. Systems that do survive are described by MP if they satisfy Equations (5) and (6), but they may implement only trivial QRFs. Hence we agree with the deflationary position advocated by Montero (2017), though for more technical reasons.

This universal requirement that awareness be supported by QRFs yields somewhat counter-intuitive predictions of minimality in basal organisms:

*Prediction 1:* Moving in 3d space does not require a QRF for 3d space, and hence does not require experiencing 3d space. *E. coli* chemotaxis provides an example. *E. coli* has a 1d spatial QRF: its body axis, with (mainly) anterior chemoreceptors and (mainly) posterior flagella. Directed “approach” motion is along this axis; undirected “tumbling” re-orients this axis randomly in the 3d “lab” frame of an observer equipped with a 3d QRF. *E. coli* has no known means of computing the relative angle between its pre- and post-tumbling linear motion, i.e. it has no known 3d QRF; hence tumbling appears to implement a 3d random walk (Wadhams and Armitage 2004). Colonial microbes living in planar mats, on the other hand, can potentially use differential cell–cell or cell–substrate interactions to distinguish left from right (Jauffred *et al.* 2017) and hence establish a 2d QRF; microbes inhabiting multi-species 3d mats with vertical division of labor may have 3d QRFs (Prieto-Barajas *et al.* 2018). Differentiated cells of multicellular eukaryotes clearly employ such QRFs (e.g. Bajpai et al. 2021); interestingly, multi-axis morphology correlates with the presence of neurons and appears to be directly instructed by neural signaling (Fields *et al.* 2020). How the representation of 3d space in migrating cells is coupled to the representations of cell- or extra-cellular surface characteristics, bioelectric and morphogen gradients, and other morphogenetic signals remains a central question in developmental biology (Grossberg 1978; Pezzulo and Levin 2015).

*Prediction 2:* Successful interaction with an object does not require a QRF that identifies that object, and hence does not require experiencing that object. *E. coli* mating provides an example: the mating pilus extends randomly in 3d space, and is tipped by an adhesin of unknown specificity (Cabezón *et al.* 2015). Commonplace lateral gene transfer (LGT) between members of distant microbial lineages (Robbins *et al.* 2016) suggests that mating without mate detection is routine in the microbial world; viral-mediated gene transfer and direct uptake of nucleic acids from the environment provide even more extreme examples. Communal microbes such as *Myxococcus xanthus* that differentiate kin from nonkin even below the species level, however, appear to have sophisticated, though yet uncharacterized, QRFs for other organisms (Muñoz-Dorado *et al.* 2016; Thiery and Kaimer 2020). Fungi that engage in differential anastomosis appear similarly equipped (de la Providencia *et al.* 2005). Cell-type identification QRFs appear to be implemented in part bioelectrically in multi-species microbial mats (Humphries *et al.* 2017; Yang *et al.* 2020); the ubiquitous use of bioelectric signaling in fungi suggests that this may be the case for fungal cell-type QRFs as well.

*Prediction 3*: Successful causation does not require a mechanism for detecting causation, and hence does not require experiencing causation. Hunting swarms of *M. xanthus* kill and eat other microbes, but appear to have separate, noncommunicating detection systems for prey species and edible prey components (Thiery and Kaimer 2020). Hence they have no way of causally associating the killing of prey with the subsequent availability of edible prey components.

These can be summarized by the following, which recognizes the key role of memory in enabling the experiences of space, objects, and causation:

*Prediction 4:* Having a memory does not require a QRF for linear time, and hence does not require experiencing time or retrievable memory. All organisms have genomes that record their phylogenetic history, but they have no mechanism for reading their previous genomic states. The genome does not, therefore, function as an internal QRF for linear time. This applies, in particular, to us, although we can employ the genomes of other organisms as *external* linear time QRFs (Kumar 2005).

If this last prediction is correct, microbes and perhaps other organisms lacking linear time QRFs may live in a “continuous present” characterized mainly by chemoreception (“taste”) and stress (see below). Their sensory capabilities may change radically via LGT or other mechanisms, but they cannot “notice” such changes.

Biological memories exist at multiple scales (Fields and Levin 2018). Characterizing the scales at which memories are encoded, and the QRFs that enable them, emerges as a central task for any theory of consciousness that assumes MP. Bioenergetic studies may provide a content-independent means of approaching these questions, as discussed below.

### Experienced memories are encoded on boundaries

Actions by an organism on its environment are, in the language of Fig. 1, preparations of an MB state |B> that its environment observes. A swimming bacterium, e.g. prepares a new environmental state by expending energy to change its location. In Friston’s (2013) terms, this is active inference: acting on the environment to change its state alters, in consequence, the subsequent environmental states observed by the active system.

Actions are, by definition, thermodynamically irreversible: they transfer classical information, via B, from the organism to its environment. Transfers of classical information across boundaries such as B are, moreover, the *only* thermodynamically irreversible events in MP. As with the requirement for QRFs to enable awareness, this is a consequence of MP’s quantum theoretic foundation. The state |*A*> is not, in general, assumed to be separable; hence its Schrödinger evolution is time-reversible and incurs no energetic cost (Bennett 1982). Information can only be retrievable at some later time if it is irreversibly and hence classically encoded. Hence retrievable memories can, in MP, only be encoded on boundaries such as B. As experiencing a memory as such requires an ability to compare two experiences—either two memories or a memory and a current state—it requires retrieval. Any experienced memory must, therefore, be both encoded on, and retrievable via an appropriate QRF from B.

This fundamental thermodynamic constraint has immediate consequences for biological architecture and for the structure of QRFs for linear time:

*Prediction 5:* All retrievable biological memories are stigmergic. Beginning with bacteria (Gloag *et al.* 2015), biological systems ubiquitously employ stigmergic memories (Heylighen 2016). This is not a surprising observation to be explained, but rather an empirical confirmation in MP. The idea that the experiencing mind “extends” (Clark 1998) into the environment via stigmergic memory is a direct consequence of quantum theory.

**Figure 3.**
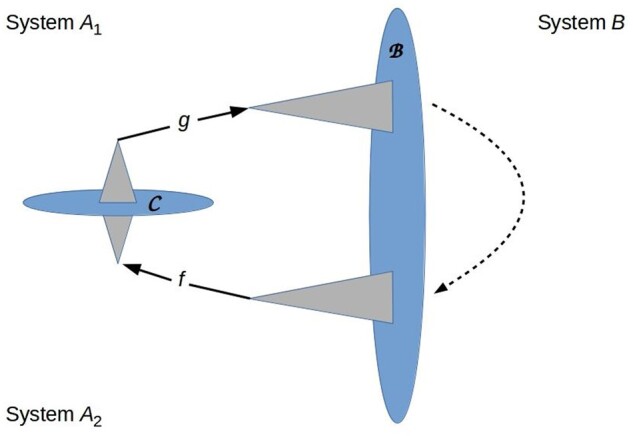
Cartoon representation of a system *A* with an internal boundary C and hence a separable state |*A*> = |*A*_1_>|*A*_2_>. Again the relationships depicted are topological, not geometric. Triangles represent QRFs; *f* and *g* are internal informational states. Information flow across C is bidirectional by Equation (5); information also flows through the environment (dashed arrow). The communication loop is closed, generating positive Φ (Oizumi *et al.* 2014).

The stigmergic nature of memory can be reconciled with the experience of memory as an internal, private phenomenon only if the agent *A* is compartmentalized by internalizing part of B to provide an internal boundary C on which classical information can be encoded. This internal boundary imposes a separability condition |*A*> = |*A*_1_>|*A*_2_> as shown in Fig. 3; *A*_1_ becomes part of the “environment” of *A*_2_ and vice-versa. The interaction between *A*_1_ and *A*_2_ can be written in the form of Equation (5); hence information flow across C is bidirectional classical communication between the components *A*_1_ and *A*_2_. If we view *A_2_* as implementing perception and *A*_1_ as implementing action, this communication, together with the environment’s response, forms a closed loop. Hence we have:

*Prediction 6:* Internal awareness requires internal boundaries. Any system *A* capable of experiencing internal memories, in particular, has a separable internal state and positive integrated information Φ. Internal memories are built into all systems that qualify as conscious in IIT (Oizumi *et al.* 2014, see especially Fig. 19) and are the basis for such systems having Φ > 0. Here, we see this as a consequence of quantum information theory.

As discussed above in connection with MBs, this prediction links separability in its quantum-theoretic sense of state distinguishability with separability in its classical sense of separation by a boundary: the internal boundary C functions as an MB that encodes classical information and imposes conditional independence. It provides for a general expectation that living systems will be compartmentalized by internal boundaries on which classical information can be encoded. Because MBs limit, as well as enabling, information transfer, it also predicts a systematic inability to determine the source of a memory. In particular:

*Prediction 7:* Compartmentalized systems cannot determine the sources of their encoded memories. Systems can be expected to behave as if memories they encode reflect their own past experience, whether they do or not. Studies of memory transplantation (Pietsch and Schneider 1969) and false-memory induction (Ramirez *et al.* 2013) in nonhuman animals provide mechanistic support for this prediction (see Levin 2020 for additional examples and discussion), as does the psychology (Henkel and Carbuto 2008) and neuroscience (Straube 2012) of false-memory induction in humans.

Organisms record memories as messages to their future selves. Neither the mechanism that recorded a memory, the internal or environmental events that induced recording, nor the context in which the recording occurred are discoverable, however, when the memory is later retrieved. This fundamental uncertainty about the sources of memories can be seen as a consequence of a more general uncertainty about whether QRFs are shared, either by distinct observers at a single time, or by a single observer across time. This question of QRF sharing is provably finite Turing undecidable (Fields *et al.* 2021).

The compartmentalization in Fig. 3 can be arbitrarily generalized:

*Prediction 8:* Experiential complexity scales with internal compartmentalization. Evolution “discovered” the benefits of internal compartmentalization ca. 3.5 billion years ago with the development of microbial biofilms exhibiting differential exposure to the open environment and division of metabolic labor (Stal 2012). The organelles of eukaryotic cells, including internal membrane complexes such as the Golgi apparatus, are canonical intracellular compartments. Multicellularity is the most common form of macroscopic compartmentalization, but is not required; examples such as *Acetabularia* (Schweiger 1969), glass sponges (Leys 2016), and syncitial fungi (Roper *et al.* 2015) all illustrate complex functional compartmentalization within single giant cells.

This prediction is clearly in line with the expectations of IIT, and again provides a physical basis for these expectations.

Cellular compartments and intercellular boundaries are, from a biological perspective, dynamic structures that must be preserved through metabolic activity. This requirement can be stated abstractly:

*Prediction 9:* Memory stability scales with the frequency of read/write cycles. The stabilization of classical bit values by repeated cycles of preparation followed by observation is called the “quantum Zeno effect” (Misra and Sudarshan 1977); the probability that the state remains stable is proportional to the frequency of observations. Hence memory decay—“forgetting”—is predicted whenever memories are not routinely accessed. This is broadly observed across phylogeny. Repair systems for nucleic acids and degradation systems for proteins provide molecular-scale examples.

Rewriting classical information costs free energy, i.e. requires metabolism as discussed below. This requirement for read/write cycles suggests that compartmentalization plays a key role in the implementation of linear time QRFs:

*Prediction 10:* Any ordered sequence of separate memories together with a comparison function can serve as a linear time QRF. Trajectories, including looming, are the simplest linear time QRFs; in the limit, they may support only sequential comparisons and hence only distinguish “then” from “now.” Insects are capable of at least short-sequence linear time perception; spiders are capable of longer sequence perception (Japyassú and Laland 2017). Merely executing a fixed action pattern does not require *experiencing* linear time, although it clearly requires an internal clock. Molecular cell-cycle clocks are as old as life, and circadian clocks are as old as cyanobacteria (Johnson 2004); both are highly conserved across phylogeny (Doherty and Kay 2010). Possessing a molecular clock does not, by itself, enable time perception.

*Prediction 11:* Time and object/feature identity are duals. Perceiving a trajectory requires perceiving an object or feature executing that trajectory; conversely, motion perception is the basis of object identity (Fields 2011). Whether insects or relatively low-complexity vertebrates are aware of objects as such or only features of their environments is unclear; bees at least have robust spatial memory and feature perception, and may recognize objects as such (Chittka 2017), while fish appear to recognize conspecifics as distinct objects (Sovrano *et al.* 2018). Spiders are capable of robust object perception and object-directed planning (Japyassú and Laland 2017), as are cephalopods (Mather 2019), birds and mammals.

Environmental objects and features that are experienced as having stable identities are, to close the circle, the canonical bearers of stigmergic memories. Without perceptible object identity, time is not perceptible and hence memories cannot be recognized as such. Only an organism capable of experiencing linear time and objects or features with stable identities can experience memories. Such an organism inhabits a rich and meaningful *umwelt*, whether its ontology easily relates to our own or not.

### The free-energy costs of irreversibility induce coarse-graining and attention-switching

With the understanding of QRFs developed above, we can return to the question of bioenergetics, see why living systems can have only limited awareness, cognitive capacity, and memory, and understand the tradeoffs between these.

The dynamics described by Equation (5) explicitly conserves energy: β*^A^T^A^* = β*^B^T^B^*. Hence some fraction of the bits transferred from *B* to *A* must be “burned” by *A* to supply the free energy required to irreversibly encode classical information (Fields and Glazebrook 2020a). These free-energy supplying bits are not available as input to any QRF (Fields *et al.* 2021). As no QRF can read the values encoded by these bits, their values are irrelevant and indeed “invisible” to *A*. They can, therefore, be considered random for *A*, i.e. to constitute environmental “noise” or in thermodynamic terms, a heat bath. As noted above, however, the source of this noise is quantum, not classical; there is no third system injecting noise into the bipartite *A-B* interaction (see Fields *et al.* 2021, for further discussion).

A central question of biology is: how can organisms maintain their phenomenal complexity with such small free energy budgets? The answer is alluded to above: *quantum* computational complexity, implemented by unitary Schrödinger evolution, is energetically free (Bennett 1982). Classical, i.e. observable or experienceable computational complexity is expensive, ln2 *k_B_T* per bit. Hence we have:

*Prediction 12:* Organisms only require the energy needed to maintain their *classical* states. As seen above, these are encoded on either exterior or intercompartmental boundaries. An organism’s energy budget must, therefore, supply the free energy needed to maintain, via Zeno-effect read/write cycles, the classical states of their compartment boundaries. Nonboundary states can remain quantum, evolve unitarily, and consume no free energy. Only the inputs and outputs of these quantum computations are classically encoded, all on boundaries.

Clearly not all boundary-localized processes are classical; electron-transport processes operating in the THz range could consume a cell’s entire energy budget if fully classical. The technical difficulty of observing nonclassical behavior in such systems (e.g. Cao *et al.* 2020) is not surprising. All of our observational outcomes are classical by definition; observing quantum coherence requires observing expectation violations in probability distributions over recorded classical events (e.g. Mermin 1993). This suggests that an indirect approach to quantitating nonclassicality in biological systems is required. From the above considerations, stable memories provide a quantitative lower limit on classicality, while the cellular energy budget provides an upper limit. Determining what states a cell or organism commits free energy to maintain, i.e. what the set points for homeostasis/allostasis are, may be the best approach to estimating the net classicality of biologically encoded information.

Biological encodings of classical information have lower limits of 1–2 nm in radius, e.g. the size of a typical protein active site (Liang *et al.* 1998) or a gap-junction channel (Sosinsky and Nicholson 2005), and about 200 fs in time, e.g. the response time of rhodopsin to photons (Wang *et al.* 1994). Cellular response times, even for bioelectric responses, are orders of magnitude larger and involve much larger areas. This loss in resolution is a consequence of bioenergetics:

*Prediction 13:* Actionable classical encodings are coarse-grained. Actionability requires irreversible encoding as in Fig. 3. The free energy to support this encoding must come from B, and hence must consume some of the bits encoded on B as fuel. The information encoded by these bits is irreversibly lost to *A*; hence all of *A*’s irreversible encodings are coarse-grained.

Any system *A* that encodes information irreversibly is, therefore, faced with a choice that its computational architecture must resolve: the tradeoff between preserving information via memory and losing information due to coarse-graining. A flexible solution to this tradeoff is to devote memory resources to only some input information, i.e. to the results computed by only some QRFs, allowing these selected results to be recorded at higher resolution while recording others either at low resolution or not at all. Hence we have:

*Prediction 14:* Living systems in complex, dynamic environments will evolve attention-switching systems. Attention has long been associated with consciousness (Engel and Singer 2001) and attention allocation is one of the main functions of competition for access to the workspace in GNW models (Dehaene and Naccache 2001), whether formulated in terms of the “rich club” (Sporns 2013), “connective core” (Shanahan 2012) or “giant component” (Wallace 2005) of the larger network. Indeed the “self” has been described as a working model of attention allocation (Graziano and Webb 2014; see also below). In humans, attention can drive entirely illusory object perception (Ongchoco and Scholl 2019), consistent with active-inference models of Bayesian-precision allocation (Kanai *et al.* 2015). Here, we see a requirement for active attention as a consequence of the thermodynamic requirements of classically encoding information.

The approach/avoid switching in simple chemotactic systems such as *E. coli* provides a basal model of the switch between active exploration and expectation revision at the heart of active inference theory (Friston 2010, 2013). Such models suggest that every such switch is governed by a reference value set by some QRF. Dorsal/ventral attention system switching in humans (Vossel *et al.* 2014) is sensitive to a vast array of expectations, the reference values of which are unknown and in at least some cases subject to considerable individual variation. Salience assignments driving both exploratory and reactive behavior are, in particular, highly dependent on individual experience, strongly coupled to the core self-representation and the reward system, and subject to variation in both prosocial and pathological directions (Uddin 2015). Much of ethology can be viewed as the comparative study of salience. Understanding how QRFs that regulate salience vary across phylogeny will be a major step toward answering the “what is it like?” question in a systematic way.

## Resource Usage, Interoception, and the Self

The considerations above reinforce the obvious point that acquiring and monitoring the usage of free-energy resources is one of the core functions of any living system. A second core function, equally necessary for the maintenance of homeostasis/allostasis, is damage control. Within the MP framework, these core functions are supported by a QRF that encodes the set points that serve as the overall homeostatic attractor. This QRF defines the “self” in MP.

*Prediction 15:* The “self” comprises three core monitoring functions, for free-energy availability, physiological status, and organismal integrity, and three core response functions, free-energy acquisition, physiological damage control, and defense against parasites and other invaders. These will be found in every organism. Indeed they are found even in *E. coli*, which has inducible metabolite acquisition and digestion systems (Jacob and Monod 1961), the generalized “heat shock” stress response system (Burdon 1986), and restriction enzymes that detect and destroy foreign, e.g. viral DNA (Horiuchi and Zinder 1972). All of these responses act to restore an overall homeostatic setpoint, i.e. an expected nonequilibrium state; hence they can all be viewed as acting to minimize environmental variational free energy or Bayesian expectation violation (Friston 2010; 2013).

Specialized molecular pathways for the core functions of the self are supplemented by specialized intercellular communication pathways in multicellular organisms and by inter-individual communication pathways in social organisms, e.g. eusocial insects (Robinson 1992) or humans. Networks of neurons support feeding, locomotion (a primary stress response) and defense already in Cnidarians (Bosch *et al.* 2017) and at least feeding and locomotion in Ctenophores (Moroz 2015); these functions become far more complex in bilaterian animals, particularly in active animals including arthropods, cephalopods, and vertebrates (Liebeskind *et al.* 2017), and form the basis of “core consciousness” in Damasio’s (2000) framework.

Considerable evidence now indicates that interoception in humans, and so presumably in mammals generally, employs a predictive coding mechanism (Hohwy 2013; Seth 2013) and is strongly coupled to the core self-representation and the salience network via the insula—cingulate—orbitofrontal loop (Craig 2010; Uddin 2015; Seth and Tsakiris 2018). This predictive coding system manages homeostasis/allostasis across the scale hierarchy from cells to organ systems (Corcoran and Hohwy 2019) and couples interoception to exteroception and proprioception (Barrett and Simmons 2015; Seth 2013; Barrett 2017; Seth and Tsakiris 2018). Intriguingly, emotional and stress responses, core components of the self, are highly sensitive to gut microbiome activity in humans and other animals (Vuong *et al.* 2017; Sarkar *et al.* 2018). As all multicellular eukaryotes have obligate symbiotic microbiomes (hence are “holobionts”; Gilbert 2014), one can expect that microbial contributions to stress detection and response are universal.

The thermodynamic costs of memory impose a particular restriction on the self, which predatory eukaryotic unicells such as *Paramecium*, animals, and quite possibly plants solve by weighting the less-certain future lower in resource priority than the (by default assumed to be) more-certain past:

*Prediction 16:* Organisms bias their “cognitive light cones” (Levin 2019), their representational and computational boundaries of concern or goal-directedness, toward the past. Memory, in other words, takes precedence over planning. The extent to which food caching, cooperative hunt organization, and other future-directed activities by nonhuman animals provides evidence of deliberative, explicit planning remains controversial (Bayne *et al.* 2019), with some arguing that all forms of imaginative “mental time travel” are human-specific (Suddendorf and Corballis 2007). It is, however, clear that explicit planning requires classically encoded representations of future events and so competes for resources with memory. As planning also requires explicit memory, it cannot win this competition.

The free-energy costs of mental time travel in either direction constrain it to an “off-line” activity when organisms are faced with rapid change that requires high-resolution perception and action. Such constraints also apply to the real-time representational costs of the self. Hence we can predict:

*Prediction 17:* Increasing real-time response requirements will disrupt encoding of the self-representation. This is observed in humans in “flow” states (Csikszentmihályi 1990), in highly automated, expertise-dependent activities including most social interaction (Bargh and Ferguson 2000; Bargh *et al.* 2012), and in experimental manipulations in which fatigue in various modalities affects cognitive performance (Massar *et al.* 2018). The extent to which nonhuman animals are able to apply “theory of mind” reasoning to themselves, and hence maintain a metacognitive self-representation, remains controversial (Martin and Santos 2016).

The picture that emerges from these energetic considerations is of a self-representation with critical functions and deep evolutionary roots (Levin 2020), but with only limited and transient exposure to awareness via explicit encoding. This is broadly consistent with the constructive views of the self outlined by Blackmore (2002), Chater (2018), Graziano and Webb (2014) among others.

## Context Change Drives Evolution, Development, and Learning

Psychology in the 20th century was consumed with controversies pitting evolution (“Nature”) against learning (“Nurture”), with developmental processes straddling the conceptual gap in between. The development of robust machine learning algorithms, the advent of evo–devo (Müller 2007; Carroll 2008), the recognition that evolution itself can be viewed as a learning process (Power *et al.* 2015; Watson and Szathmáry 2016; Kouvaris *et al.* 2017), and in particular, the scale-free applicability of active inference models (Friston 2013; Friston *et al.* 2015; Fields and Levin 2020b,c; Kuchling *et al.* 2020) have done much to deconstruct these distinctions. As a scale-free approach in which any lineage can also be considered an individual (Fields and Levin 2020c), MP treats evolution, development, and learning as instances of a common mechanism. It distinguishes two nonoverlapping cases: gradual alterations in the relative contributions of QRFs or components of QRFs to outcomes, and saltatory changes in QRFs themselves.

*Prediction 18:* Context changes drive QRF changes. Here, we define context changes strictly: For a fixed set {*o_i_*} of observables, a context change *x* → *y* has occurred if the probability distributions Prob(*o_i_*|*x*) and Prob (*o_i_*|*y*) are well defined but the joint distribution Prob(*o_i_*|x∨y) is not (Kochen and Specker 1967; Dzhafarov *et al.* 2017). Here, “observables” are by definition classifiers, each of whose components consist of event/(condition, context)/valuation triples (Fields and Glazebrook 2020c; Fields *et al.* 2021). Under these conditions, QRF change in response to context change is required to maintain Bayesian coherence (Fields and Glazebrook 2020c).

Context switches are hard to measure, as doing so requires predicting what “background” variables are relevant in fact to how an event is processed, a prediction problem that is in general intractable (Dietrich and Fields 2020). Saltatory changes in event representations, e.g. canonical “Aha!” moments (Kounios and Beeman 2015), may reflect context-driven QRF changes. Basal systems may provide uniquely manipulable windows into such processes: e.g. Pezzulo *et al.* (2020) have recently suggested that bioelectrically driven saltatory morphological changes in planaria may employ the same underlying mechanism as saltatory changes in episodic memories in mammals.

## Conclusions

Quantum theory has had a long and somewhat disreputable association, dating back at least to von Neumann (1932), with the science of consciousness. A dominant concern, beginning at least with Wigner (1961) and extending through Penrose (1989), Schwartz *et al.* (2005), and Hameroff and Penrose (2014) to Georgiev (2020) has been to employ quantum theory to establish an ontological foundation for a science of consciousness. Our interest here has not been ontological, but rather empirical: to derive as much as possible from the simple assumption that consciousness involves information exchange subject to the constraints of quantum information theory. We have shown that the MP framework that follows from this assumption allows many of the key features of consciousness to be understood as simple, scale-independent consequences of thermodynamics.

In direct contrast with strict Cartesianism, MP holds that we can better understand our own awareness by understanding the awareness of our more basal cousins. Our homeostatic/allostatic drives and the mechanisms that satisfy them are phylogenetically continuous with those of prokaryotic unicells including *E. coli*. Our concepts and categories are implemented by QRFs playing the same roles, and satisfying the same basic requirements, as the Che-Y system regulating bacterial chemotaxis. The tradeoffs that we implement, and adjust in real time, between perception, memory, and planning are tradeoffs that have been explored and adjusted in niche-specific ways by all organisms throughout evolutionary history. We can take advantage of these fundamental mechanistic similarities to design theoretical and experimental paradigms that reveal and assess scale-free properties of consciousness in both natural and engineered systems.
